# Cysteamine-Gold Coated Carboxylated Fluorescent Nanoparticle Mediated Point-of-Care Dual-Modality Detection of the H5N1 Pathogenic Virus

**DOI:** 10.3390/ijms23147957

**Published:** 2022-07-19

**Authors:** Kaliannan Durairaj, Duc Duong Than, Anh Thi Viet Nguyen, Hak Sung Kim, Seon-Ju Yeo, Hyun Park

**Affiliations:** 1 Zoonosis Research Center, Department of Infection Biology, School of Medicine, Wonkwang University, Iksan 54538, Korea; kmdurairaj@gmail.com (K.D.); ducduong27189@gmail.com (D.D.T.); anh.nguyenviet@vnuk.edu.vn (A.T.V.N.); 2 College of Pharmacy, Wonkwang University, Iksan 54538, Korea; hankidad@wku.ac.kr; 3 Department of Tropical Medicine and Parasitology, Department of Biomedical Sciences, College of Medicine, Seoul National University, Seoul 03080, Korea

**Keywords:** cysteamine gold-europium nanocomposite, lateral flow immune assay, dual-mode assay, avian influenza virus, monoclonal antibody

## Abstract

Globally, point-of-care testing (POCT) is the most preferable on-site technique for disease detection and includes a rapid diagnostic test (RDT) and fluorescent immunochromatographic strip test (FICT). The testing kits are generally insufficient in terms of signal enhancement, which is a major drawback of this approach. Sensitive and timely on-site POCT methods with high signal enhancement are therefore essential for the accurate diagnosis of infectious diseases. Herein, we prepare cysteamine-gold coated carboxylated europium chelated nanoparticle (Cys Au-EuNPs)-mediated POCT for the detection of the H5N1 avian influenza virus (AIV). Commercial nanoparticles were used for comparison. The spectral characteristics, surface morphologies, functional groups, surface charge and stability of the Cys AuNPs, EuNPs, and Cys Au-EuNPs were confirmed by UV-visible spectrophotometry, fluorescence spectrometry, transmission electron microscope with Selected area electron diffraction (TEM-SAED), Fourier-transform infrared spectroscopy (FTIR) and zeta potential analysis. The particle size distribution revealed an average size of ~130 ± 0.66 nm for the Cys Au-EuNPs. The Cys Au-EuNP-mediated RDT (colorimetric analysis) and FICT kit revealed a limit of detection (LOD) of 10 HAU/mL and 2.5 HAU/mL, respectively, for H5N1 under different titer conditions. The obtained LOD is eight-fold that of commercial nanoparticle conjugates. The photo luminance (PL) stability of ~3% the Cys Au-EuNPs conjugates that was obtained under UV light irradiation differs considerably from that of the commercial nanoparticle conjugates. Overall, the developed Cys Au-EuNPs-mediated dual-mode POCT kit can be used as an effective nanocomposite for the development of on-site monitoring systems for infectious disease surveillance.

## 1. Introduction

Globally, numerous analytical devices have been used to detect infectious diseases, including enzyme-linked immunosorbent assay (ELISA), immunofluorescence, real-time reverse transcription-polymerase chain reaction (rRT-PCR), serological testing, the immunowall device, POCT, and nucleic acid sequencing [1,2,3,4,5,6,7]. Although such methods provide powerful approaches for the detection of infectious diseases, limitations include the complexity, high cost, essential training, suitable instruments, and high-quality samples required [1,2,8]. The POCT approach can deliver results within ~15 to 20 min, and is thus the most preferable on-site detecting technique. POCT includes two types of analysis: colorimetric (visual detection by rapid diagnostic test (RDT)); and fluorescence (ultra-violet light detection using portable medisensor by fluorescent immunochromatographic strip test (FICT)) analysis [9].

Over the past few decades, europium nanoparticles (EuNPs) and colloidal gold nanoparticles (AuNPs) have been used in FICT and RDT, respectively, to detect infectious diseases. However, the major weaknesses of colorimetric or fluorescence analysis (single-mode detection approaches), and specifically EuNP-mediated FICT, are that personnel are required to operate the portable sensors, and that FICT shows weak signals when analyzing biological samples (such as blood, urine, or feces). Improvements have been attempted by equipping the devices with noble metal nanoparticles (such as Silver (Ag), Platinum (Pt), Iridium oxide (IrO_2_), Iron (II, III) oxide (Fe₃O₄), Selenium (Se)), and quantum dots (QDs). However, the specific issues have not yet been fixed [10,11,12,13,14].

Recent research has started to focus on developing POCT kits that are based on dual-mode on-site detection, which requires multiple noble nanoparticles or QDs. The metals such as Au-Ag, Au-Pt, Pt-Pd, and QDs have been used in RDT kits; however, so far improvements to the LOD have only been obtained using these methods with single-mode approaches [15,16,17,18,19,20,21]. Although our group has previously developed RDT devices for single-mode detection [22], several commercial EuNPs, such as florescent dyes [23], aliphatic amine latex beads [24], CdSe/CdS/ZnS QDs, and latex coated CdSe/CdS/ZnS QDs [17], show good sensitivity (93%) and specificity (100%). Our group has previously focused on enhancing the LOD with single-mode detection. However, the use of the single-mode approach in the development of the RDT and FICT kits for detection of the AIV H5N1 subtype is limited.

Herein, our aim is to develop a dual-mode RDT kit that uses cysteamine-AuNPs with carboxylated EuNPs for the detection of the H5N1 virus. The strong stability and inverse sensitivity of cysteamine-AuNPs (Cys-AuNPs) renders them as suitable alternatives to the citrate-mediated AuNPs [25]. The amine and thiol groups on the Cys-AuNPs generate a positive charge, while the efferent fluorescent material comprising EuNPs has led to their use in microplate and microchip readers [26]. The disadvantages of using the previously reported fluorescent dye and QD-mediated POC test kits include problems with photo-oxidation and single-mode detection. Based on these issues we intend to develop a dual-mode approach. Until now, no study has reported on the use of a Cys-Au-coated carboxylated EuNPs (Cys Au-EuNPs)-mediated LFIA (lateral flow immunoassay) kit for the dual-modality detection of the H5N1 subtype AIV [27]. In addition, we introduce novel H5N1 subtype-specific monoclonal antibodies and prove the performance of dual-modality detection (FICT and RDT).

## 2. Results

### 2.1. Development of Monoclonal Antibodies

**Figure 1:**
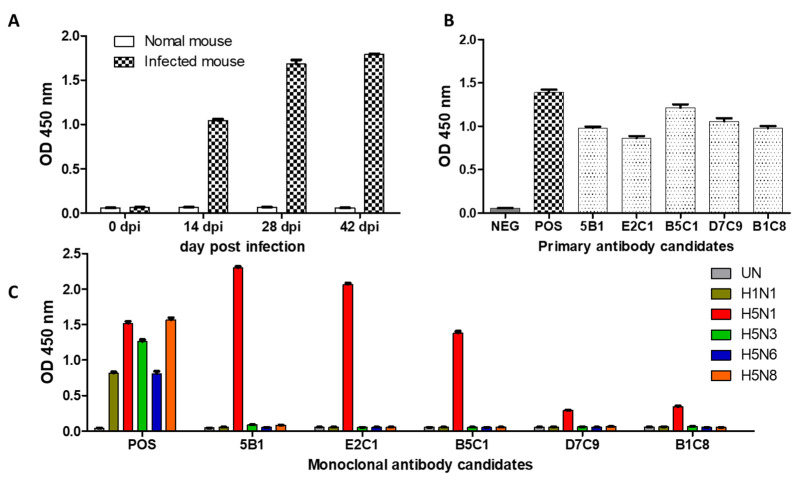
Development of H5N1 (2.3.2.1c) specific antibodies. (**A**) Mice were immunized with inactivated H5N1-RG virus. BALB/c mice were immunized every two weeks. Sera were collected to evaluate titer of antibodies against the H5N1 (2.3.2.1c) virus; (**B**) Cell supernatant with primary antibody candidates was collected and screened by ELISA; (**C**) Cell supernatant with monoclonal antibodies was evaluated by indirect ELISA.

The immunization of mouse models by footpad is an important, safe, and effective method that has already been used to produce many kinds of monoclonal antibodies [28,29,30]. In this study, BALB/c mice were immunized with HA (hemagglutination assay) titers of 1000 HAU/mL inactivated whole H5N1-RG virus emulsified with an equal volume of adjuvant at two-weekly intervals. The sera were collected from the immunized mice every 2 weeks and the antibody titer tested by indirect ELISA (Figure 1A). Seven days after the final immunization, the spleen and lymph nodes were harvested and fused with SP2/0 cells. The hybridoma cell lines that survived after 2 weeks of treatment with HAT and HT were screened by ELISA to obtain those that could produce monoclonal antibodies. The primary cell clones were then cultured in 24-well plates and the capability confirmed by indirect ELISA (Figure 1B). Based on the results, five cell clones were selected for continuous culture in a T75 cell culture flask, so that cross-checking for other AIV subtypes (such as H1N1, H5N1, H5N6, H5N3, and H5N8) could be conducted. The results indicated (Figure 1C) the highest titer for the two clones, 5B1 and E2C1, neither of which showed a cross reaction with the other subtypes.

### 2.2. Characterization of Nanoparticles

**Figure 2:**
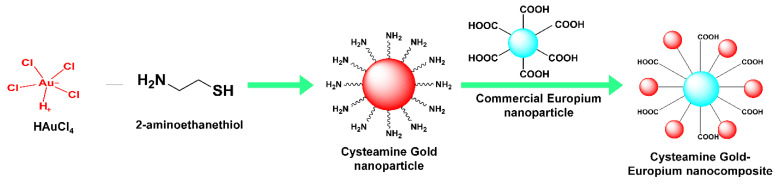
Schematic diagram detailing the preparation of Cys Au-Eu Nps.

The Cys Au-Eu Nps were prepared and applied to the LFIA kit for dual-modality detection. The preparation of the Cys Au-Eu Nps is shown schematically in Figure 2.

**Figure 3:**
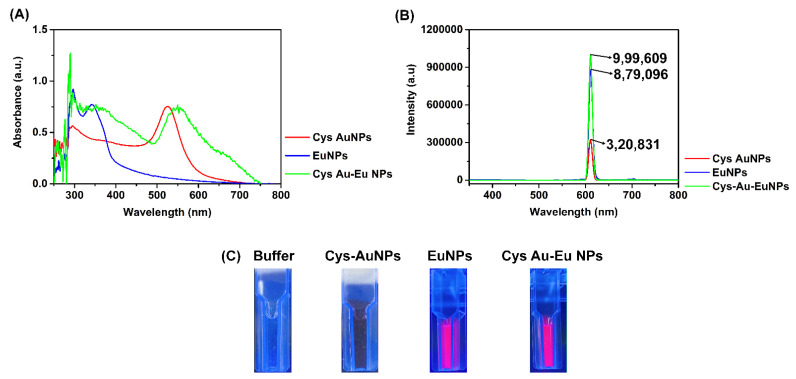
(**A**) UV-Visible Spectrum analysis and (**B**) fluorescence spectrum analysis of Cys AuNPs (Red line), EuNPs (Blue line), and Cys Au-Eu NPs (Green line); (**C**) Fluorescence images of buffer and nanoparticles are taken under UV exposure.

First, the Cys AuNPs were synthesized using a one-pot synthesis reaction. Once integrated, the synthesized Cys AuNPs were confirmed based on the reduction of auric ions (Au^3+^-A^0^) to AuNPs and the color of the reaction, which changed from pale yellow to ruby red [31,32]. In addition, the maximum (λ_max_) and the strong localized surface-plasmon resonance (LSPR) band peaked at 520–530 nm (visible range), which is a result of the colloidal Cys AuNPs in the solution. The commercially obtained europium nanoparticles were also characterized by the presence of a LSPR band at 280–370 nm (ultra-violet). Lastly, the preparation of the Cys Au-Eu NPs was performed as a single-pot reaction with PBS buffer. The reactions of the amine (-NH) and carboxyl groups (-COOH) were well bonded, based on the ionic bond interactions and the presence of the synthesized Cys Au-Eu Nps was revealed, based on the LSPR absorbance peaks at both the ultra-violet (280–400 nm) and visible (510–590) wavelengths (Figure 3A).

The photo-luminance (PL) was characterized by an excitation wavelength of 610 nm and an emission range of 200 nm to 800 nm in a fluorescence spectrophotometer. Cys AuNps are naturally fluorescent [33] with a confirmed intensity (320,631 a.u.). The major goal was to coat the fluorescent material onto the AuNPs to enhance the PL intensity. Accordingly, the prepared Cys Au-Eu NPs revealed a strong intensity (999,609) as compared to that of the commercial EuNPs (879,096 a.u.) (Figure 3B). The optical properties of the Cys Au-Eu Nps were analyzed under UV light and are disclosed in Figure 3C. The UV exposure images prove the strong intensity of the Cys Au-Eu NPs.

**Figure 4:**
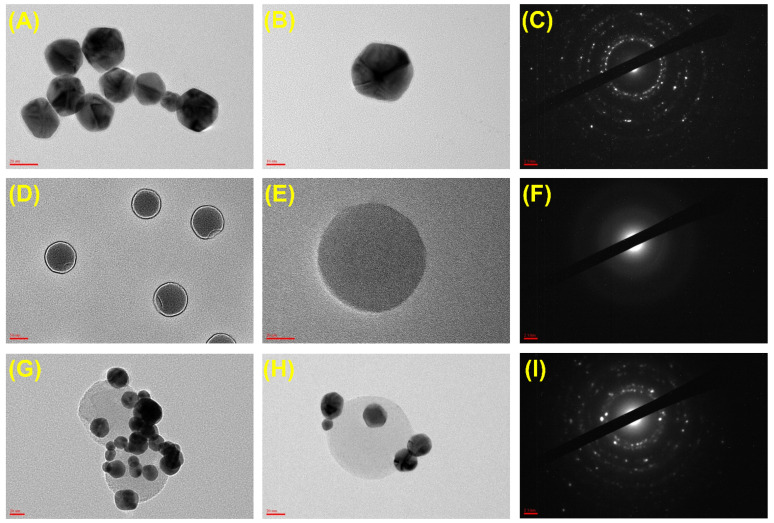
TEM with SAED pattern analysis (**A**–**C**) Cys AuNPs; (**D**–**F**) Commercial EuNPs; (**G**–**I**) Cys Au-EuNPs. Scale bar—(**A**,**D**,**G**) 20 nm; (**B**,**E**,**H**) 10 nm; (**C**,**F**,**I**) 2 1/nm.

The surface morphology and the average size distribution of the nanoparticles were observed by TEM analysis. The Cys AuNPs (Figure 4A–C) and EuNPs (Figure 4D,E) were almost spherical and well-dispersed. The synthesized Cys Au-Eu NPs were also spherical but were aggregated (Figure 4G,H). The surface morphologies and the nanoparticle coatings were, thus, as predicted in accordance with the schematic expectations (Figure 2). The SAED (selected area electron diffraction) analysis showed irregular patterns for the Cys AuNPs (Figure 4C) and regular banding (Figure 4F) for the EuNPs. The irregular and regular SAED patterns generated confirmed that an adequate coating of the Cys Au-Eu Nps occurred (Figure 4I).

**Figure 5:**
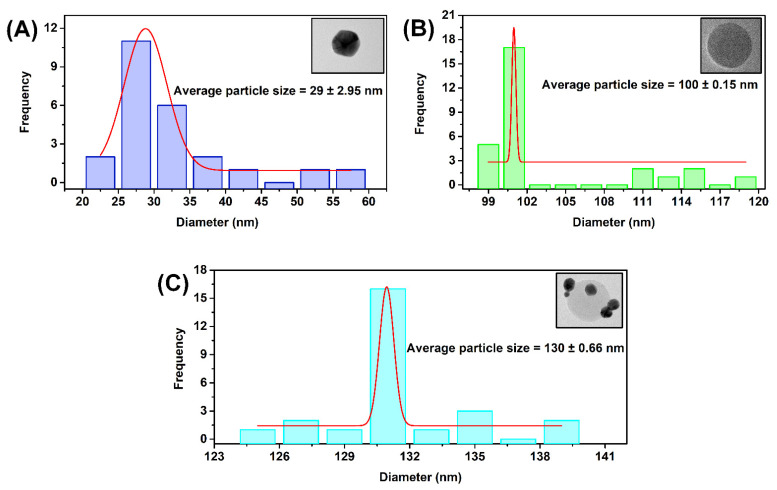
Particle size distribution of (**A**) Cys AuNPs; (**B**) Commercial EuNPs; (**C**) Cys Au-EuNPs.

According to the particle size distribution histogram (Figure 5A–C), the average sizes of the Cys AuNPs, EuNPs, and Cys Au-Eu Nps are estimated to be 29 ± 2.95 nm, 100 ± 0.15 nm, and 130 ± 0.66 nm, respectively. The average diameters of the nanoparticles are almost the same according to the approximations obtained, using the Haiss and Bangs equations. The Haiss calculation for the particles before and after coating indicated Cys AuNps concentrations of 5.514 × 10^11^ nps/mL (Cys AuNps) and 7.5 × 10^9^ nps/mL (Cys Au-Eu Nps), respectively, while values of 1.803 × 10^13^ nps/mL (EuNPs) and 5.45 × 10^3^ nps/mL (Cys Au-Eu Nps) were obtained for the EuNps before and after coating, respectively, via the Bangs equation. Details of the calculations are given in Figure S2 (Supplementary Materials) [34,35]. In addition, the functional group of those nanoparticles was analyzed by Fourier Transform Infrared spectroscopy (FTIR) and those results proved the formation of the ammonium and carboxyl groups on the surface of the EuNPs and Cys Au-Eu Nps (Figure S3, Supplementary Materials) [36]. Additionally, the surface charge of the Cys Au-Eu NPs was confirmed in negative charge, and it was stable in higher pH ranges. So, the Cys Au-EuNPs are highly suitable for further conjugation and the detailed results are presented in the Supplementary Materials (Figures S4 and S5, Supplementary Materials).

### 2.3. Development of a Cys Au-Eu NPs Conjugate Linked RDT and FICT Assay

**Figure 6:**
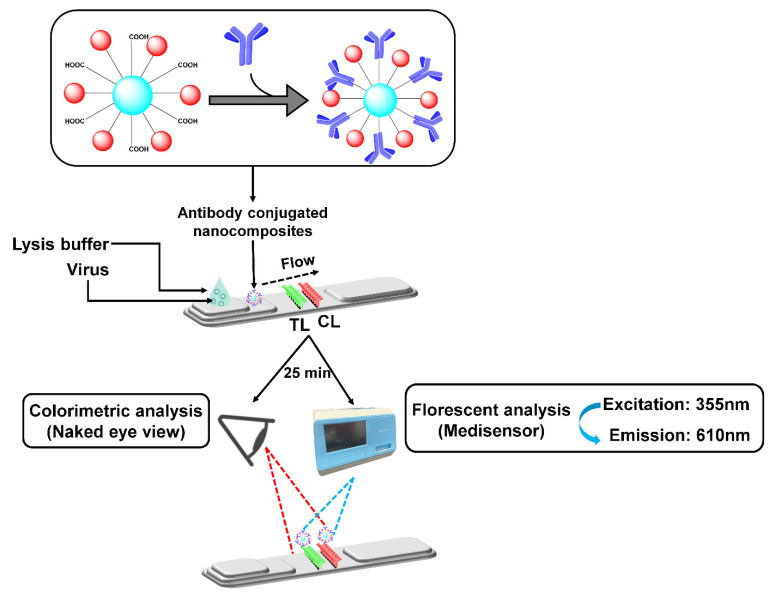
Schematic diagram showing preparation of RDT and FICT kit assays.

The antibody conjugation of the Cys Au-EuNPs was tested using RDT and FICT assays, with the images obtained by the naked eye and medisensor analysis, respectively (Figure 6). To achieve this, the RDT and FICT assays and nanoparticles conjugates were loaded onto a conjugation pad and dried for 15 min at 37 °C. A lack of reaction between the conjugated material and the monoclonal antibody (mAb) on the TL (test line) indicates the absence of the virus, whereas a reaction indicates that the virus is present. The CL (control line) strips were coated with anti-mouse IgG, which recognizes the sample virus NP antibody (Ab) in the conjugates. Thus, the LFIA kits were used as the tests. Both the colorimetric and fluorescence signals were noted within 25 min of placing the virus sample onto the strip. The TL/CL values indicate the amount of virus in the sample. A comparison of the RDT and FICT was achieved, using commercial AuNPs and EuNPs conjugates as a positive control.

**Figure 7:**
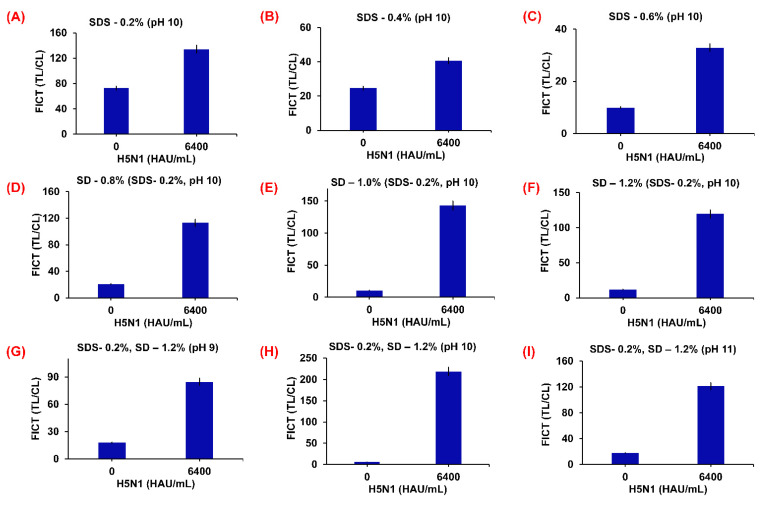
Optimization of lysis buffer for Cys Au-Eu nanocomposite-FICT. Various concentrations of SDS (0.2, 0.4, and 0.6%), SD (0.8, 1.0, and 1.2%) at different pH (9, 10, and 11) were tested in basic lysis buffer (0.1 M Tris, 0.1 M EDTA, and 1% Triton X-100). Basic lysis buffer was dissolved at three different SDS concentrations (**A**–**C**); 0.2% SDS was dissolved in lysis buffers of different SD concentrations (**D**–**F**) at pH 10; 0.2% SDS and 1.2% SD was dissolved in lysis buffers at different pH (**G**–**I**).

**Figure 8:**
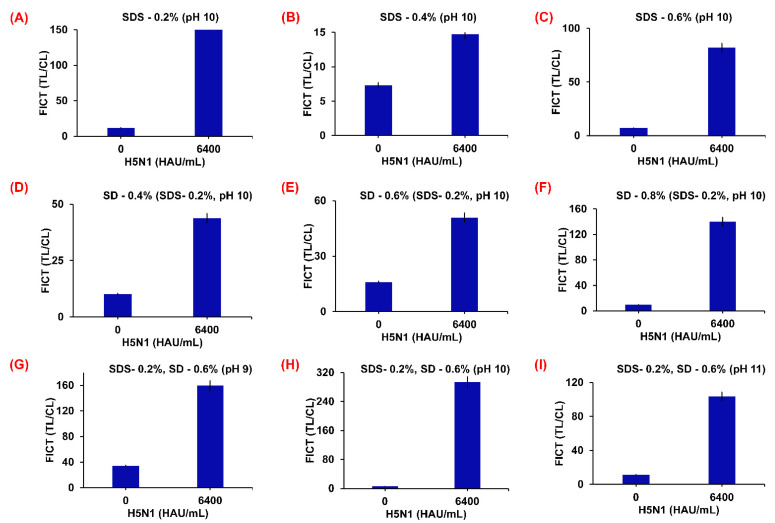
Optimization of lysis buffer for EuNPs-FICT. Various concentrations of SDS (0.2, 0.4, and 0.6%), SD (0.8, 1.0, and 1.2%), and different pH values (9, 10, and 11) (0.1 M Tris, 0.1 M EDTA, and 1% Triton X-100) were tested in basic lysis buffer. Buffer was dissolved at three different SDS concentrations (**A**–**C**); 0.2% SDS was dissolved in lysis buffers of different SD concentrations (**D**–**F**) at pH 10; 0.2% SDS, and 1.2% SD were dissolved in lysis buffers at different pH (**G**–**I**).

To prepare the H5N1 virus at a titer concentration of 6400 HAU/mL for both the RDT and FICT assay requires lysis buffer optimization. This means that the diagnostic kit assay essentially needs a lysis buffer to prevent cross-reactivity at the TL on the strip. The optimization of the RDT and FICT assay lysis buffer was achieved using different SDS (0.2, 0.4, and 0.6%) and SD (0.8, 1.0, and 1.2%) combinations at different pH (9, 10, and 11) (Figure 7 and Figure 8). The optimized lysis buffer conditions were thus used to detect the H5N1 virus: for Cys Au-EuNPs-RDT (0.1 M Tris-HCl, 0.1 M EDTA, 0.4% Triton X-100, 0.6% SDS, 0.6% SD, pH 10); for AuNPs-RDT (0.1 M Tris-HCl, 0.1 M EDTA, 0.6% Triton X-100, 0.6% SDS, pH 10); and for EuNPs and Cys Au-EuNps-FICT (0.1 M Tris-HCl, 0.1 M EDTA, 1% Triton X-100, 0.2% SDS, 1.2% SD, pH 10). The raw data describing the optimization of the FICT assay lysis buffer are presented in Figures S6 and S7 (Supplementary Materials).

### 2.4. Performance of Cys Au-Eu NPs Conjugates in RDT and FICT Kits

After the development of the complete RDT and FICT assays, the spiked H5N1 virus that was diluted two-fold was accomplished for both the RDT and FICT assays with optimized lysis buffer. The RDT LOD was analyzed by colorimetry (naked eye). Likewise, the FICT LOD was obtained using the TL/CL values, that were calculated using the previously described equations [37]. The limit of blank (LOB) is the highest apparent concentration of analytes that is predicted when the replicates of a blank sample containing no analytes are tested. The LOD is the lowest concentration of analytes that is likely to be reliably distinguished from the LOB for which detection is considered viable:

(1) $$\mathrm{LOB}=\mathrm{mean}\;\mathrm{blank}+1.645*\left(\mathrm{SD}\;\mathrm{blank}\right)\;$$

(2) $$\mathrm{LOD}\;=\mathrm{LOB}+1.645*\left(\mathrm{SD}\;\mathrm{of}\;\mathrm{low}\;\mathrm{virus}\;\mathrm{titer}\right)$$

**Figure 9:**
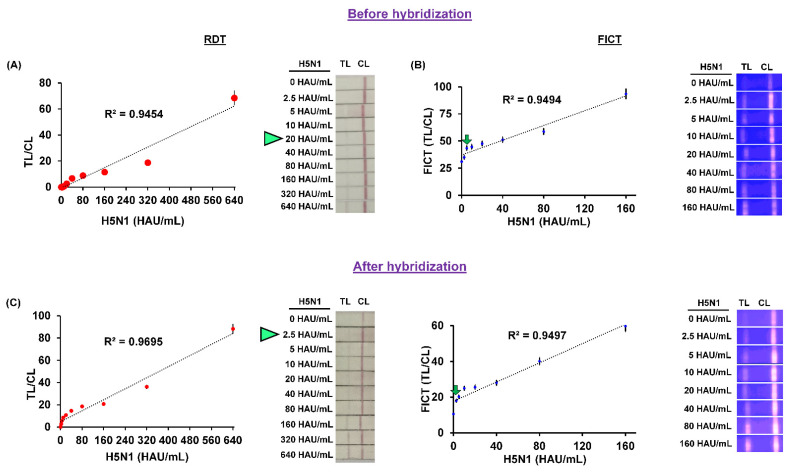
Comparison of nanomaterial performance in FICT and RDT. Two-fold serially diluted influenza A virus H5N1 subtypes were tested with (**A**) commercial gold nanoparticles—RDT; (**B**) Europium nanoparticles (Eu NP)-FICT; and (**C**) Cys Au-Eu nanocomposites (Cys Au-Eu NPs), both RDT and FICT. The measured FICT values were plotted on a graph and fluorescence images of Eu NP- and Cys Au-Eu NPs are shown in the left panel. The linear range for FICT using the fluorescent material conjugates was determined and the data (*n* = 3) are shown as mean ± SD.

The LOD for the RDT kit using conjugated commercial AuNPs to detect H5N1, based on naked-eye analysis, was determined at 20 HAU/mL. Likewise, the LOD for Cys Au-EuNPs was calculated as 2.5 HAU/mL. The strip TL/CL values for the LOD were analyzed as 5 HAU/mL for FICT, using EuNP conjugates. Similarly, the LOD of Cys Au-EuNPs was confirmed as 2.5 HAU/mL. The results for the synthesized Cys Au-Eu NPs, RDT, and FICT indicate a high performance compared with the commercial nanoparticle conjugates (AuNPs and EuNPs) (Figure 9).

**Figure 10:**
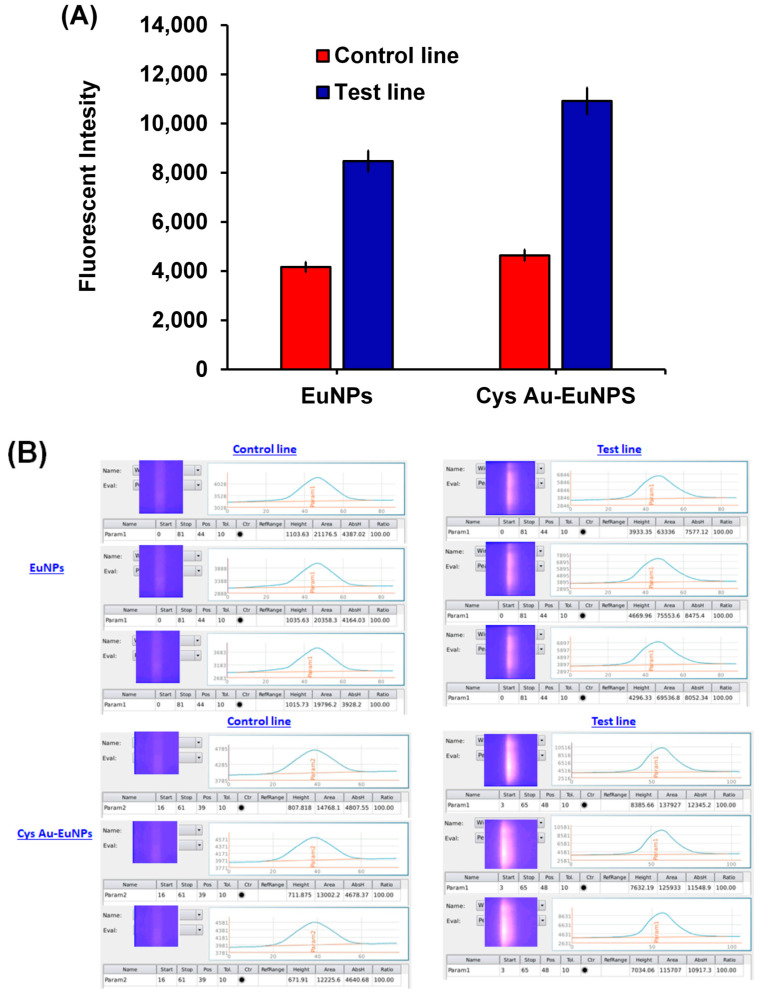
(**A**) Fluorescence intensity for CL and TL of EuNPs and Cys AU-EuNPs; (**B**) Raw data describing medisensor information.

**Figure 11:**
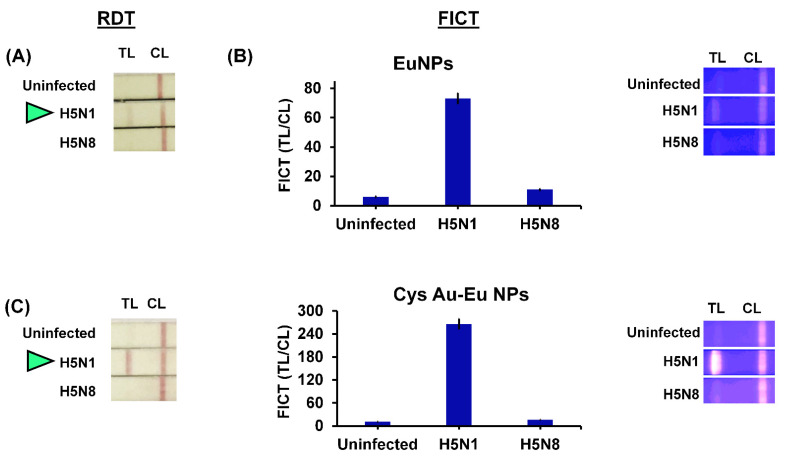
Comparison of the performance of nanomaterials tested for different H5 subtype viruses by FICT and RDT. (**A**) Commercial AuNPs—RDT; (**B**) Eu NP-FICT; and (**C**) Cys Au-EuNPs—RDT and FICT.

Figure 9 also indicates an improvement in the fluorescence intensity of the CL and TL for the nano-conjugates. The raw data from the medisensor-mediated FICT and RDT are presented in the Supplementary Materials(Figures S8–S12). A comparison analysis of the RDT and FICT with another H5 subtype virus (H5N8) was performed at a titer concentration of 1500 HAU/mL. Interestingly, the results showed that the nanoparticle conjugates achieved satisfactory results. The detailed results are presented in Figure 10 and Figure 11, and the raw data used for the FICT analysis are given in Figures S13 and S14 (Supplementary Materials). Due to the small number of clinical samples, the clinical experiments could not be performed using RDT and FICT.

### 2.5. Stability of Cys Au-Eu NPs Conjugates under UV Irradiation

**Figure 12:**
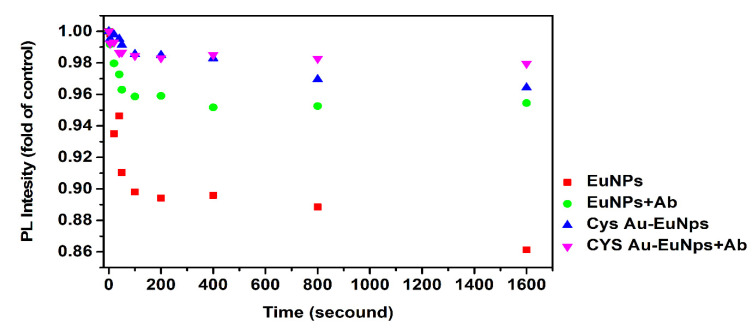
PL intensity visualization of changes in Eu NPs, Eu NP + Ab conjugate, Cys Au-EuNPs, and Cys Au-EuNPs + Ab conjugates in response to UV irradiation normalized to the control (value of UV exposure over 0 s).

The use of fluorescent nanomaterial conjugates in LFIA requires that the photophysical, photochemical, and other PL properties are tested under UV-light irradiation. The experiment was set up based on our team’s optimized protocols, with slight modifications [17]. The EuNPs, EuNPs+mAb conjugate, Cys Au-EuNPs, and Cys Au-EuNPs+mAb conjugates were placed in a storage buffer under UV irradiation for 20 min and then analyzed using a fluorescence spectrophotometer. Figure S14 (Supplementary Materials) shows the PL intensity of the EuNPs, EuNPs+mAb conjugate, Cys Au-EuNPs, and Cys Au-EuNPs+mAb conjugates over 20 min. The results show that the Cys Au-EuNPs were more stable, in terms of fluorophore production. The raw data images detailing the fluorescence of nanomaterials and conjugates are shown in Figure S15 (Supplementary Materials). Figure 12 reveals the photo-oxidation performance, with the different time interval PL intensity values normalized using the control value at 0 s UV exposure. The result for the PL intensity of the EuNPs and EuNPs-mAb conjugates decreased by up to 96% and 86%, respectively. The PL intensity of the commercial materials varied by up to 10%. Correspondingly, only a slight variation was observed in the PL intensity of the Cys Au-EuNPs, and Cys Au-EuNPs+mAb conjugates (3%). The presence of DO (dissolved oxygen) in the buffer solution was found to decrease the PL and photo-oxidation of the Cys Au-EuNPs, although this effect was minimal [38].

## 3. Discussion

The WHO reported that more than 400 people died worldwide from infection with the HPAIV H5N1 subtype in 2021 [39]. The main risk factor for human infection is both direct and indirect exposure to contaminated environments (such as poultry or markets), although there is no evidence to suggest that HPAIV has been transmitted via these routes [40]. Controlling the circulation of AIV in poultry is essential to reducing the risk of human infection. According to the information released by the WHO, the RT-PCR diagnosis of AIV has been standardized [41]. However, the availability of specific H5N1 AIV LFIA kits is limited [7], and the kits that can be obtained require significant amounts of bio-conjugation (nanomaterial/QD conjugate with antigens and antibodies). The single nanomaterial/QD-mediated LFIA kits are familiar and have been extensively explored, using either colorimetric or fluorescence analysis [27,42]. The modern nano-biotechnologists wish to develop a dual-mode approach, based on the LFIA kits; however, until now, no evidence focusing on AIV detection using nano-bio hybrid materials has been obtained, and no H5N1 subtype-specific LFIA kits have been produced [27].

Table 1 describes the single-mode detection approach for the AIV H5 subtypes. The materials used have proven stability and optical properties. Currently, there are no kits available on the market for H5 AIV detection. Based on the reports, fluorescence detection has a significantly higher capacity for detecting such viruses compared to colorimetric detection, and can provide results within an average of 30 min. However, the single-mode detection approach has disadvantages, such as cross-reactivity, high cost, and the requirement for more than one sample. Because of these limitations, modern nano-biotechnologists are aiming to develop a dual-mode approach.

In the present study, we prepared nanocomposites and accomplished metal selection, based on the information that was obtained previously. The Cys AuNPs have a potentially positive charge with strong stability, and Wai et al. demonstrated the electrostatic interaction of Cys AuNPs at different pH ranges, which they termed a “critical re-dispersion concentration” [25]. Similarly, earlier reports suggest that the EuNPs have surface-functionalized carboxylated groups with strong stability that easily attach to amines and thiol ligands through covalent bond interactions [48]. The EuNPs obtained from Bangs Laboratories are well-established fluorescence chelates, and our research group has previously developed EuNP-mediated FICT kits for the detection of AIV [22,26,49,50]. Nonetheless, both of the types of nanoparticle have thus far been used only in single mode detection approaches.

At present, on-site diagnosis using dual mode detection is limited. This study involved the development of a rapid diagnostic system, using Cys Au-EuNPs that were conjugated with H5N1 specific monoclonal antibodies, to detect AIV H5N1 subtypes. The nanomaterial and the antibodies are conjugated through covalent bond interactions, and the prepared nanomaterial is used for a dual-mode detection approach to on-site monitoring of AIV. The experimental results demonstrate the efficiency of the nanoparticles compared to commercial nanoparticles (AuNPs and EuNPs). Table 2 presents a summary of the results for the LOD of Cys Au-EuNPs tested with various H5N1 virus samples. Compared to the performance of other such kits, our dual-mode approach shows excellent and efficient results. The dual-mode detection of the normal swab specimens through Cys Au-EuNPs conjugates was found to produce LODs of 10 HAU mL^−1^ and 2.5 HAU mL^−1^ for RDT and FICT, respectively. Our study describes the modern on-site diagnosis of AIV H5N1 via a specific dual-mode approach that is expected to be useful for AIV surveillance and management.

## 4. Materials and Method

### 4.1. Reagents

The hydrogen tetrachloroaurate (III) (HAuCl_4_), cysteamine (NH_2_CH_2_CH_2_SH), bovine serum albumin (BSA) lyophilized powder ±96%, N-hydroxysulfosuccinimide sodium salt (Sulfo-NHS), sodium phosphate monobasic (NaH_2_PO_4_) 99.0%, sodium phosphate dibasic (Na_2_HPO_4_), 2-(N-morpholino) ethanesulfonic acid hydrate (MES hydrate) ±99.5%, trizma base 99.9%, anhydrous ethylenediaminetetraacetic acid (EDTA), sodium tetraborate decahydrate 99.5%, Triton™ X-100 BioXtra, sodium dodecyl sulfate (SDS) 98.5%, sodium deoxycholate (SD), and Tris hydrochloride (Tris-HCl) > 99% were purchased from Sigma-Aldrich (St. Louis, MO, USA). The hydrochloric acid (HCl), nitric acid (HNO_3_), and sodium borohydride (NaBH_4_) were purchased from Daejung Chemicals (Siheung-si, Korea). The PS-COOH Europium chelate was purchased from Bangs Laboratories Inc. (Fishers, IN, USA). The N-(3-Dimethylaminopropyl)-N′-ethylcarbodiimide hydrochloride (EDC) was from Thermo Scientific (Waltham, MA, USA). The goat polyclonal anti-mouse IgG was purchased from Koma-Biotech Inc. (Seoul, Korea). The conjugate pad (PT-R1) and nitrocellulose (NC) membrane (CNPF-SN12, 10 μm) were bought from MDI Membrane Technologies, Inc. (Harrisburg, PA, USA). The absorption pad (AP22, Grade 222) and sample pad (Glass Fiber, Grade 8964) were obtained from BoreDaBiotech Co. (Gyeonggi, Korea). The backing card was obtained from JNTG Inc. (Chungnam, Korea). Deionized water (DW) was used in all of the experiments.

### 4.2. Cell Fusion and Production of Hybridoma Cells

The H5N1-RG virus (HA based on A/Vietnam/14011801/2014) was incubated for 48 h at 37 °C with a final concentration of 0.2% formalin in PBS. The formalin was removed by dialysis with 1X-PBS immediately after inactivation. The inactivated virus was confirmed by three passages of culture on MDCK cells. The dialyzed samples were stored at −80 °C for use. The virus was mixed with an equal volume of Freund’s complete adjuvant (Sigma-Aldrich) and injected into 6-week-old female BALB/c mice (Daehan Bio-Link, Eumseong, Korea), which were boosted three times at two-weekly intervals. The sera collected from the immunized mice were used to evaluate the antibody titer via ELISA. The B cells from the spleen and lymph nodes of the selected mice were then isolated and fused with myeloma cells (F/0 cell line) and 50% polyethylene glycol and seeded in a 96-well culture plate. The hybridoma cells were selected following subculture in HAT (hypoxanthine, aminopterin, and thymidine) and HT (hypoxanthine and thymidine) media in a 5% CO_2_ incubator at 37 °C for 2 weeks. The supernatant was screened by ELISA when colonies were observed in the wells. After sub-cloning by limiting dilution, the suitable colonies were transferred into 75 cm^2^ cell culture flasks. To scale-up the mAb production, mAb-producing cells were intraperitoneally injected into an 8-week-old female BALB/c mouse. After 2 weeks, the mouse ascites were harvested and centrifuged at 5000× *g* for 15 min and the purified mAb was obtained from the ascites using a protein A agarose column (Amersham Biosciences, Uppsala, Sweden) [26].

### 4.3. ELISA

The viruses were diluted to 1000 HAU/mL with 50 mM bicarbonate/carbonate coating buffer (pH 9.6) (hemagglutinin assay unit) and 100 µL/well was added to a 96-well plate (Greiner CELLSTAR® 96 well plates, Seoul, Korea) and maintained at 37 °C for 2 h. The plate was washed five times with 200 μL PBS and 0.1% Tween 20 (PBS-T, pH 7.4) and then blocked with 5% non-fat dry milk at 37 °C, over 2 h. The primary antibody (5 μg/100 μL/well) and the positive control (anti-influenza A NP, 2 μg/100 μL/well) were then added to each well and incubated for 1 h at 37 °C to detect each virus subtype. The secondary antibody, in the form of horseradish peroxidase (HRP)-conjugated rabbit anti-mouse IgG (Abcam, Cambridge, UK), was then added to each well, according to the manufacture’s protocol. Stringent washing with PBS-T was performed five times to remove any nonspecific binding and 100 μL 3,3′,5,5′-tetra methyl benzidine (Sigma-Aldrich) substrate solution was added. After 10 min, 100 µL of 0.18 M sulfuric acid was added to stop the reaction. The optical density (OD) was measured at 450 nm by an ELISA plate reader [26,51].

### 4.4. Synthesis of Cysteamine Gold Nanoparticles

All of the glassware was soaked with aqua reagents (HNO_3_:HCl of 3:1 (*v/v*)), extensively rinsed with DW, and air-dried for 12 h. The Cys-AuNPs were synthesized, according to the available protocols with minor modifications [52]. In brief, 425 mL of fresh cysteamine (200 mM) was mixed with a 3.75 mL fresh HAuCl4 (2 mM) solution and stirred in the dark for 20 min, after which 1 mL fresh cold NaBH_4_ (10 mM) was slowly added under vigorous stirring for 20 min, followed by gentle stirring for 1 h. The solution was then maintained in dark conditions at 4 °C until required.

### 4.5. Preparation of Cys-Au Coated Carboxyl Fluorescent Nanoparticles

The Cys-Au coated carboxyl fluorescent nanoparticles were prepared by one-pot synthesis. Briefly, 9 mL of colloidal Cys-AuNPs were dissolved into 100 µL 1X phosphate-buffered saline (PBS, pH 7.4) with 1 mL of PS-COOH Europium chelate (diameter 100 nm) and mixed overnight at room temperature (RT). The resulting solution was washed with storage buffer (2 mM Borax, 0.15% BSA, pH 9.0) and centrifuged (27,237× *g*, 10 min). The prepared Cys-Au-coated PS-COOH europium chelate (hereafter Cys Au-EuNPs) was added to 2 mL storage buffer and the results stored at 4 °C until required.

### 4.6. Characterization of Nanoparticles

The UV-Visible spectra were recorded on a Nanodrop 2000c spectrophotometer (Thermo Scientific, WA, USA). The photoluminescence (PL) spectroscopy measurements were performed on the RF-6000 Spectro fluorophotometer (Shimadzu, Gangnam-gu, Seoul, Korea). The TEM was performed with selected area diffraction (SAED), using a JEM-2010F electron microscope (JEOL, Tokyo, Japan) with an accelerating voltage of 200 kV. For the TEM, 0.1% sample solutions were dropped onto a mesh copper grid and the solvents evaporated on a hotplate. The particle size distribution was calculated based on the TEM images, using Image J software. The functional group of nanoparticles was analyzed through FTIR spectroscopy (PerkinElmer Spectrum Version 10.5.1, Korea) and the surface charge and stability of the Cys Au-EuNPs was also confirmed through a zeta potential analyzer (ELSZ-1000 common Version 5.22/3.00, Korea).

### 4.7. Conjugation of Antibodies and Nanoparticles

The antibodies were covalently conjugated onto the nanoparticles (Cys Au-EuNPs and EuNPs) by a well-established protocol by Bangs Laboratories, with slight modifications. In brief, 20 µL of nanoparticle was added to 980 µL 50 mM MES (pH 6.1) and incubated for 1 h at 25 °C in the presence of 25 µL–5 mM EDC and 200 µL–50 mM sulfo-NHS. The excess EDC and sulfo-NHS were removed by centrifugation at 27,237× *g* for 10 min. The surface group-activated nanoparticles were mixed with 50 µL of 1 mg/mL antibody in 1000 µL–0.1 M sodium phosphate (pH 8.0) and allowed to react for two hours at RT. After centrifugation (27,237× *g* for 10 min), the nanoparticle-conjugated antibody was collected and re-suspended in 300 µL storage buffer (2 mM borax, 0.1% BSA, pH 8.0), and stored at 4 °C for FICT and RDT strip analysis.

Commercial colloidal AuNPs were used for RDT strip comparison. The absorptive conjugation method was used to conjugate the antibody with the colloidal AuNPs. In brief, 3 mL (OD (530 nm) = 1) AuNPs were centrifuged at 27,237× *g* for 10 min, after which the pellet was dispersed in 950 µL of 20 mM Tris buffer (pH 9.5); 50 µL of 1 mg/mL antibody were added, and the resulting solution was incubated for 3 h at RT. The mix was then centrifuged at 27,237× *g* for 10 min. Finally, the Au NP conjugated antibody was collected, re-suspended in 100 µL storage buffer, and stored at 4 °C for RDT strip analysis.

### 4.8. Lateral Flow Test Strips for FICT and RDT

The Lateral Flow Test Strips were prepared, as per the optimized protocol previously developed by our group (Yeo et al., 2018). The lateral flow test strips comprise four components: an absorbent pad; conjugate pad; sample application pad; and NC membrane. A schematic diagram of the lateral flow test strip is shown in Figure S1 (Supplementary Materials). The control line (CL) was coated with 0.3 mg/mL of goat anti-mouse IgG (Life Technologies) and the test line (TL) with 5 mg/mL anti-influenza H5 subtype-specific monoclonal antibodies. The membranes were then dried at 30 °C for 3 days, before FICT and RDT analysis.

### 4.9. Preparation of FICT and RDT Assays

Both of the assays were prepared, as per the optimized procedure that was previously developed by our group with minor modifications [22]. For the FICT, 4 μL of conjugate solution was diluted 40-fold, and the EuNPs-Ab conjugation and the Cys Au-EuNPs-Ab complex dropped onto a conjugate pad. The conjugate pad was then dried in an incubator for 15 min at 35 °C before immersing both the pad and the strip in a mixture containing 70 μL sample and 120 μL lysis buffer (0.1 M Tris-HCl, 0.1 M EDTA, 1% Triton X-100, optimized (SDS, SD, pH)) in a 1500- μL Eppendorf tube (E-tube) and incubated for 20 min at RT. The result was analyzed using a portable strip fluorescence reader with excitation at 355 nm and emission at 612 nm (Medisensor, Daegu, Korea). During the lateral flow, the AIV in the sample reacted with the nanoparticle-antibody and the TL/CL ratio was used to confirm the FICT assay performance.

For the RDT assay, 4 μL of conjugate solution was diluted two-fold and colloidal AuNPs-Ab conjugation and Cys Au-EuNPs-Ab complex dropped onto a conjugate pad. The conjugate pad was dried in an incubator for 15 min at 35 °C and both the pad and the strip were immersed in a mixture containing 70 μL sample and 120 μL lysis buffer (0.1 M Tris-HCl, 0.1 M EDTA, 0.6% Triton X-100, optimized (SDS, SD, pH)) in a 1500- μL Eppendorf tube (E-tube) and incubated for 30 min at RT. The same sample mixture ratio was used for the Cys Au-EuNPs; however, the lysis buffer composition differed (0.1 M Tris-HCl, 0.1 M EDTA, 0.6% Triton X-100, optimized (SDS, SD, pH)) and incubation was performed for 20 min at RT. The result was analyzed by colorimetric analysis, and the TL/CL ratio was used to confirm the RDT assay performance.

### 4.10. Stability Analysis

The stability analysis was performed as per our previous procedure [17]. The photo-oxidation experiments were performed using RT ultraviolet (UV) treatment. The PL—the related intensity of the commercial Eu NPs, Eu NP-Ab conjugate, Cys Au-EuNPs, and Cys Au-EuNPs-Ab conjugate under UV irradiation (302 nm, 220 mW cm^−2^)—was measured over 20 min using a spectrofluorophotometer (SHIMADZU, Columbia, MD, USA). The samples were diluted 200-fold to avoid saturation during measurement. The changes in the PL of the commercial Eu NPs, Eu NP-Ab conjugate, Cys Au-EuNPs, and Cys Au-EuNPs-Ab conjugates under UV irradiation were also confirmed by obtaining visual images, using a BIO-RAD Molecular Imager® ChemiDocTM XRS Imaging System.

### 4.11. Statistical Analysis

All of the data are shown as mean ± standard deviations (SD) of biological replicates and plotted using GraphPad Prism 5.0 (GraphPad, La Jolla, CA, USA).

## 5. Conclusions

In conclusion, the prepared Cys Au-EuNPs-mediated LFIA kit can be used for dual-modality detection of the H5N1 virus. This dual-mode approach significantly improves the on-site diagnosis. The use of dual signals (colorimetric and fluorescent signals) can provide reliable results in different situations. The potential surface charge and fluorescence stability of Cys Au-EuNPs can improve the LOD approximately eight-fold as compared to commercial nanomaterial conjugates. The current study revealed that the simplicity and high sensitivity of the diagnostic LFIA kit that can provide quantitative H5N1 AIV detection renders the kit promising for on-site monitoring, and this study proposes an innovative diagnostic technology for application in the biomedical field.

## Figures and Tables

**Table 1:** Single-mode detection approach for AIV H5 subtypes with LOD.

Mode of Detection	Conjugate Material	LOD	H5 Subtype Sample	Reference
Colorimetric	AuNPs	1 pg	Human serum	[43]
Colorimetric	AuNPs	100 pg	-	[44]
Colorimetric	Peptide—AuNPs	230 pg	-	[45]
Fluorescence	QD	90 pg mL^−1^	Chicken serum	[46]
Fluorescence	Peptide—EuNPs	20 HAU mL^−1^	Human nasopharyngeal specimens	[47]
Colorimetric	Commercial RDT	80 HAU mL^−1^		
Fluorescence	Peptide—EuNPs	5 HAU mL^−1^	-	
Fluorescence	Latex beads	1.25 × 10^4^ PFU mL^−1^	-	[24]

**Table 2:** Summary results for the LOD of commercial AuNPs, commercial EuNPs, and cysteamine Au-Eu nanocomposites tested in various titers of original H5N1 virus samples with lysis buffer and optimized conditions.

	Colorimetric Analysis (HAU mL^−1^)	Fluorescence Analysis (HAU mL^−1^)
Commercial EuNPs	-	5
Commercial AuNPs	80	-
Cys Au-EuNPs	10	2.5

## Data Availability

All the data presented in this study are available in this article and Supplementary Materials.

## Supplementary Materials

The following supporting information can be downloaded at: https://www.mdpi.com/article/10.3390/ijms23147957/s1.
