# Supplementary material for: Cysteamine-Gold Coated Carboxylated Fluorescent Nanoparticle Mediated Point-of-Care Dual-Modality Detection of the H5N1 Pathogenic Virus
Source: Int J Mol Sci. 2022 Jul 19;23(14):7957. doi: 10.3390/ijms23147957 (PMC9320457; doi:10.3390/ijms23147957)

Supplementary data

Cysteamine-gold coated carboxylated fluorescent nanoparticle mediated point-of-care dual-modality detection of the H5N1 pathogenic virus

Figure S1. Schematic diagram of the lateral flow test strip. TL - test line; CL - control line; NC - nitrocellulose.

Figure S2. Calculations of average diameter and nanoparticle concentrations using the Haiss and Bangs equations.

Figure S3. FTIR analysis of Cys AuNPs, EuNPs and Cys Au-EuNPs.

Figure S4. Zeta potential analysis of Cys Au-EuNPs

Figure S5. Stability analysis of Cys Au-EuNPs

Figure S6. Raw data describing antibody conjugated Cys Au-EuNPs tests conducted under various lysis buffer conditions.

Figure S7. Raw data describing antibody conjugated EuNPs tests conducted under various lysis buffer conditions.

Figure S8. Raw data describing antibody conjugated commercial EuNP FICT conducted with various H5N1 titers under optimized lysis buffer conditions.

Figure S9. Raw data describing antibody conjugated commercial AuNP RDT with various H5N1 titers under optimized lysis buffer conditions.

Figure S10. Raw data describing antibody conjugated Cys Au-EuNP FICT with various H5N1 titers under optimized lysis buffer conditions.

Figure S11. Raw data describing antibody conjugated Cys Au-EuNP RDT with various H5N1 titers under optimized lysis buffer conditions

Figure S12. Raw data describing antibody conjugated Commercial EuNps tested with different H5 subtype viruses using FICT.

Figure S13. Raw data describing antibody conjugated Cys Au-EuNPs tested with different H5 subtype viruses using FICT.

Figure S14. PL intensity of (A) EuNps, (B) EuNPs + Ab conjugate, (C) Cys Au-EuNPs, and (D) Cys Au-EuNPs + Ab conjugate in response to UV irradiation normalized to the control (value of UV exposure over 0 s).

Figure S15. Raw images of fluorescent metals and conjugates.

Figure S1.

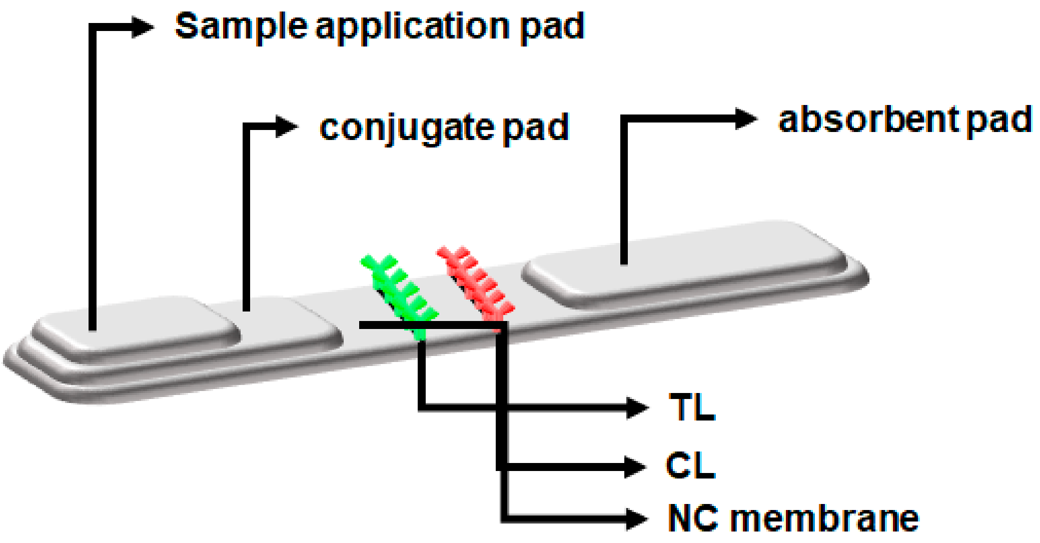

Sample application pad
conjugate pad
absorbent pad
TL
CL
NC membrane

Figure S2.

Cys AuNPs by Haiss equation

$$d = \frac{\ln\left(\frac{\lambda_{spr} - \lambda_0}{L_1}\right)}{L_2}$$

λ_{spr} = wavelength at the peak of the SPR,
$\lambda_0 = 512$,
$L_1 = 6.53$,
$L_2 = 0.0216$

**Before coating Cys AuNPs Diameter (*d*) = ~30nm**
**After coating Cys AuNPs Diameter (*d*) = ~80nm**

Calculating AuNPs concentration (N = gold nanoparticle/ml)

$$N = \frac{A_{450} X 10^{14}}{d^2\left[-0.295 + 1.36\exp\left(-\left(\frac{d - 96.8}{78.2}\right)^2\right)\right]}$$

A_{450} = absorption at 450 nm,
d = diameter of the NPs

**Before coating Cys AuNPs numbers**
N = 5.514 * 10^11 nps/mL
**After coating Cys AuNPs numbers**
N = 7.5 * 10^9 nps/mL

EuNPs by Bangs equation

**EuNPs Diameter (*d*) = ~100nm**
**Cys Au-Eu NPs Diameter (*d*) = ~150nm**

$$N = \frac{6 \times 10^{10} \bullet S \bullet \rho_L}{\pi \bullet \rho_S \bullet d^3}$$

N	=	# microspheres/mL for suspensions in water
S	=	weight % solids (for 10% solids suspension, S=10)
ρL	=	density of microsphere suspension (g/mL)
ρ_L	=	$100 \bullet \rho_S / [S(1-\rho_S) + (100 \bullet \rho_S)]$
ρ_S	=	density of solid sphere (g/cm^3)
d	=	mean diameter (µm)

**Before coating EuNPs numbers**
N = 1.803 * 10^13 nps/mL
**After coating EuNPs numbers**
N = 5.45*10^3 nps/mL

Figure S3

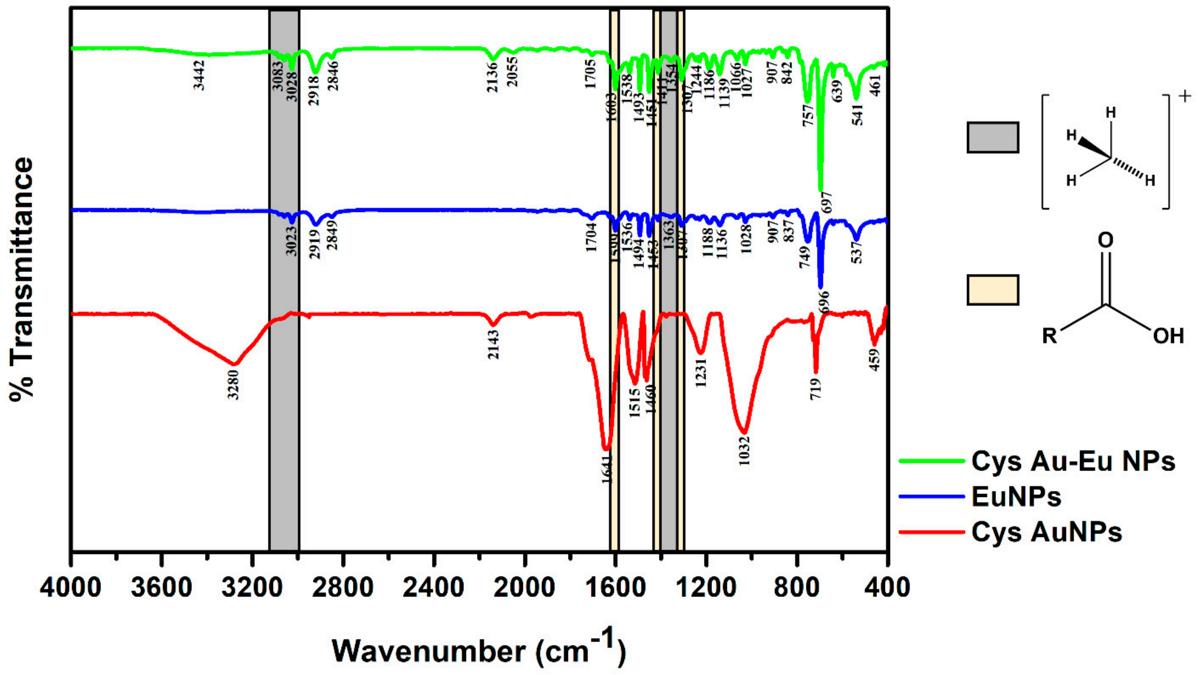

% Transmittance
Wavenumber (cm-1)
4000
3600
3200
2800
2400
2000
1600
1200
800
400
Cys Au-Eu NPs
EuNPs
Cys AuNPs
R
OH
O
H

Figure S4

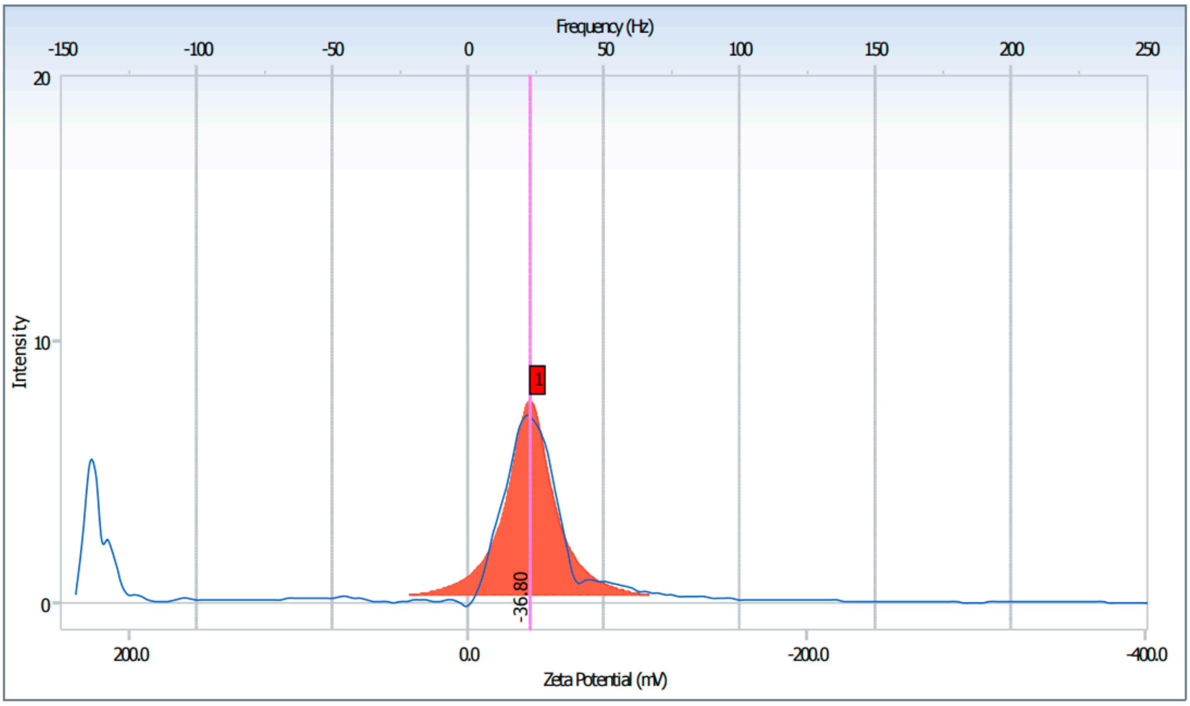

Frequency (Hz)
-150
-100
-50
0
50
100
150
200
250
20
10
0
Intensity
1
-36.80
200.0
0.0
-200.0
-400.0
Zeta Potential (mV)

Figure S5.

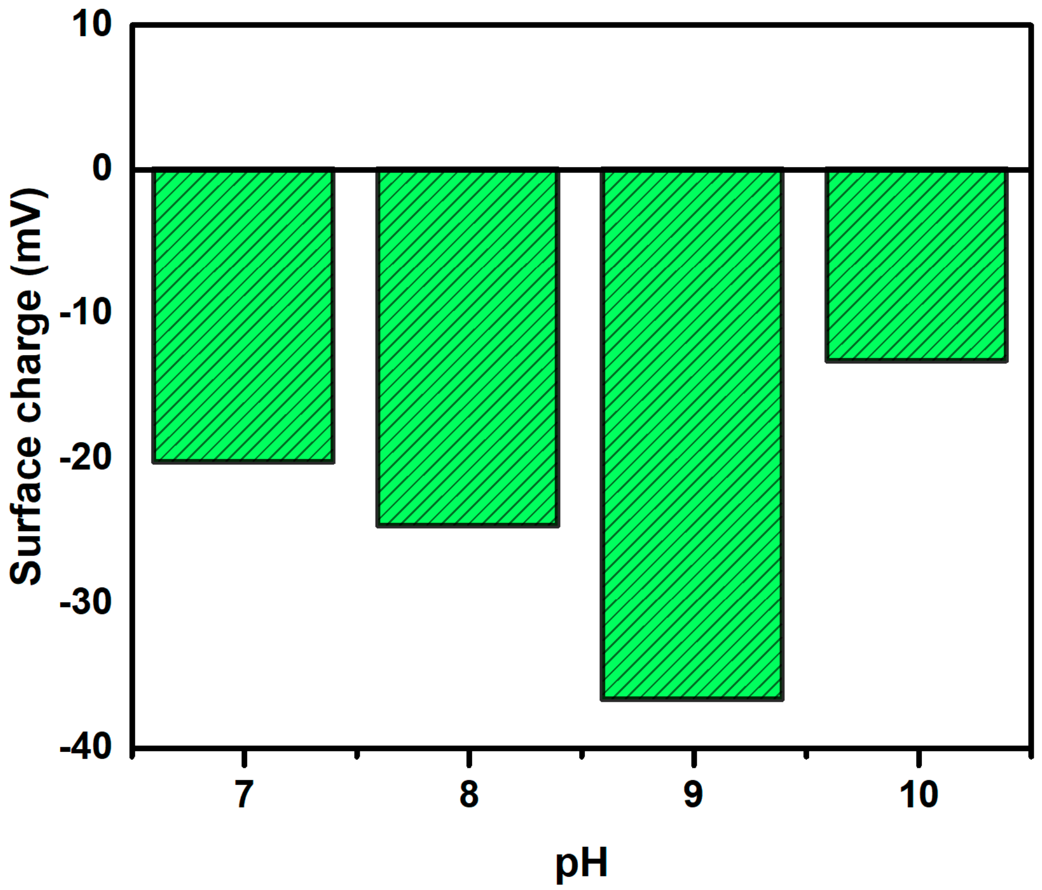

Figure S6.

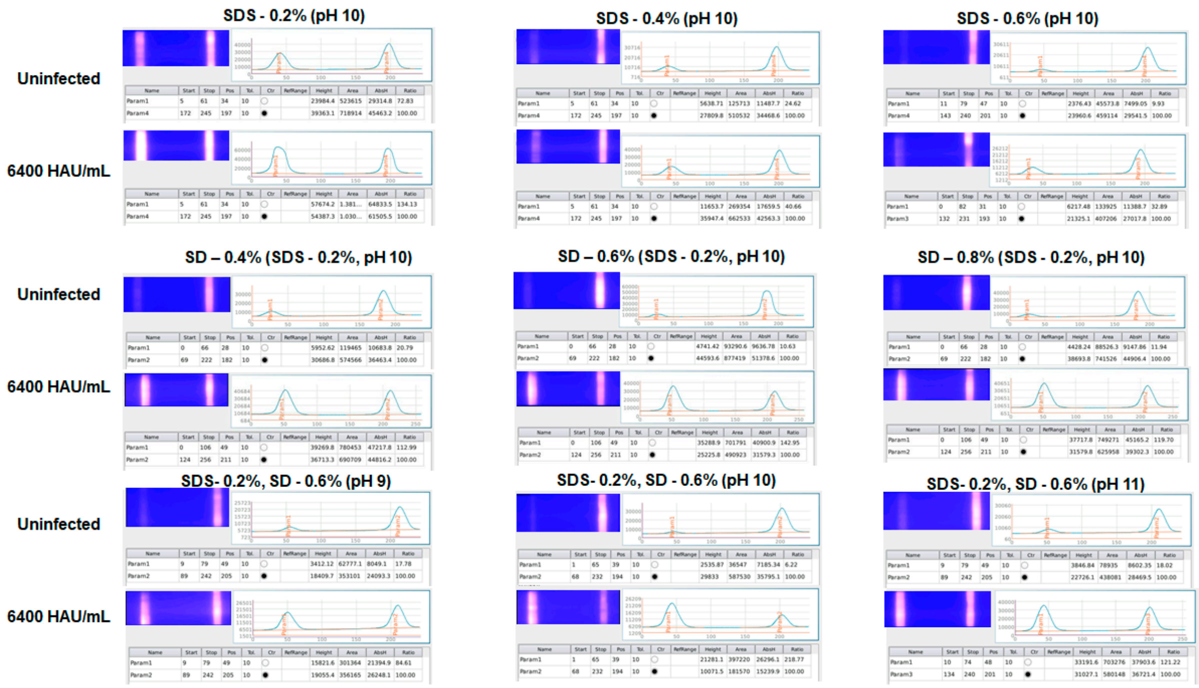

SDS - 0.2% (pH 10)
SDS - 0.4% (pH 10)
SDS - 0.6% (pH 10)
SD – 0.4% (SDS - 0.2%, pH 10)
SD – 0.6% (SDS - 0.2%, pH 10)
SD – 0.8% (SDS - 0.2%, pH 10)
SDS- 0.2%, SD - 0.6% (pH 9)
SDS- 0.2%, SD - 0.6% (pH 10)
SDS- 0.2%, SD - 0.6% (pH 11)
Uninfected
6400 HAU/mL
Uninfected
6400 HAU/mL
Uninfected
6400 HAU/mL

Figure S7.

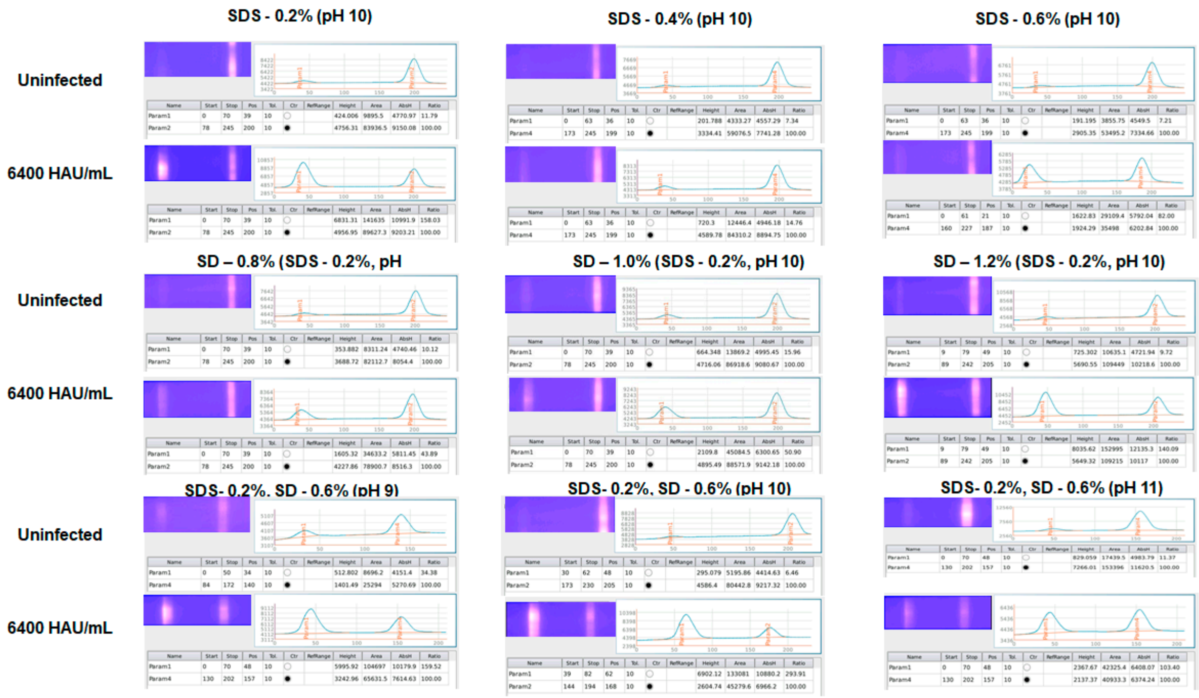
SDS - 0.2% (pH 10)
SDS - 0.4% (pH 10)
SDS - 0.6% (pH 10)
Uninfected
6400 HAU/mL
SD – 0.8% (SDS - 0.2%, pH
SD – 1.0% (SDS - 0.2%, pH 10)
SD – 1.2% (SDS - 0.2%, pH 10)
Uninfected
6400 HAU/mL
SDS- 0.2%, SD - 0.6% (pH 9)
SDS- 0.2%, SD - 0.6% (pH 10)
SDS- 0.2%, SD - 0.6% (pH 11)
Uninfected
6400 HAU/mL

Figure S8.

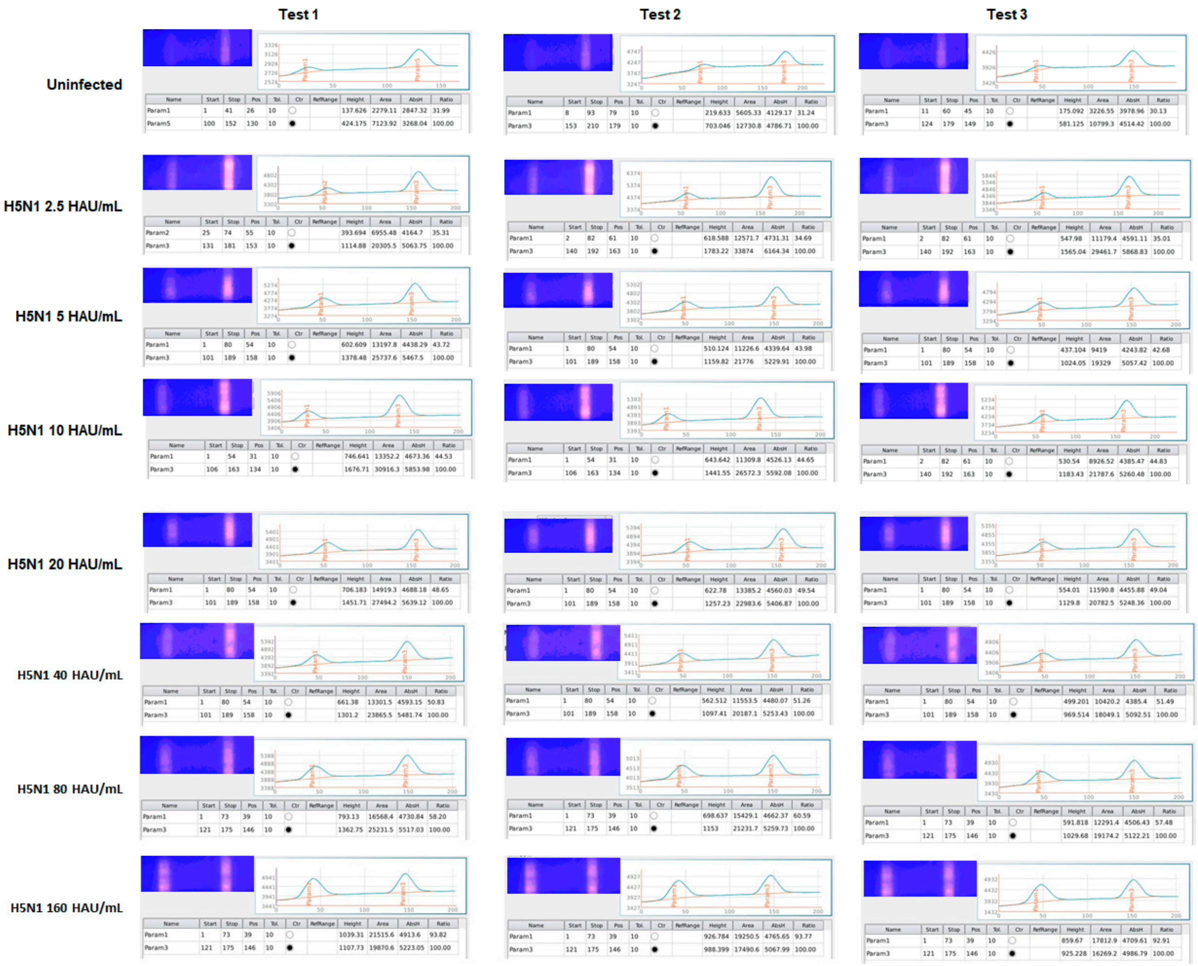

Test 1
Test 2
Test 3
Uninfected
H5N1 2.5 HAU/mL
H5N1 5 HAU/mL
H5N1 10 HAU/mL
H5N1 20 HAU/mL
H5N1 40 HAU/mL
H5N1 80 HAU/mL
H5N1 160 HAU/mL

Figure S9.

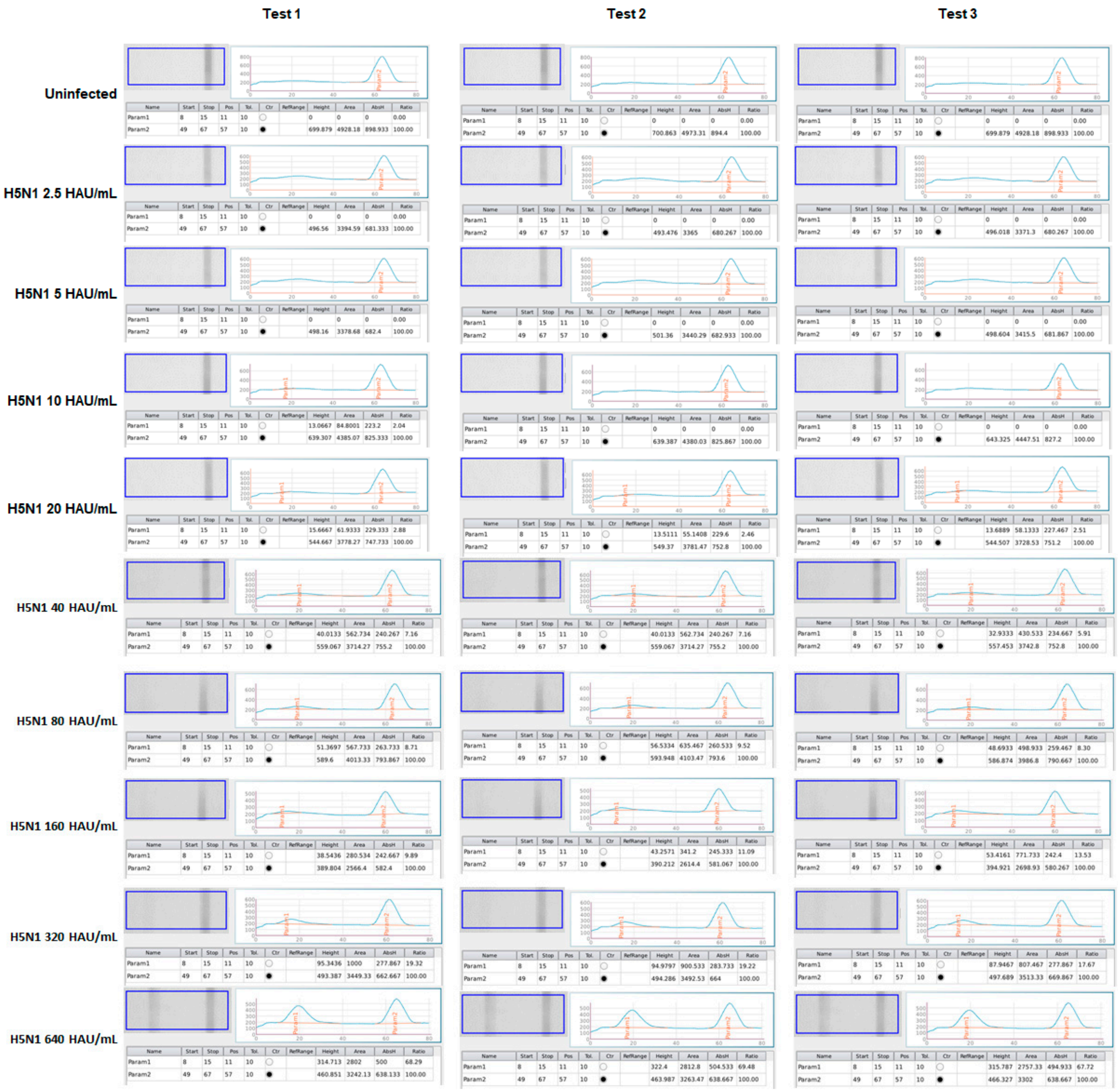
Test 1
Test 2
Test 3
Uninfected
H5N1 2.5 HAU/mL
H5N1 5 HAU/mL
H5N1 10 HAU/mL
H5N1 20 HAU/mL
H5N1 40 HAU/mL
H5N1 80 HAU/mL
H5N1 160 HAU/mL
H5N1 320 HAU/mL
H5N1 640 HAU/mL

Figure S10.

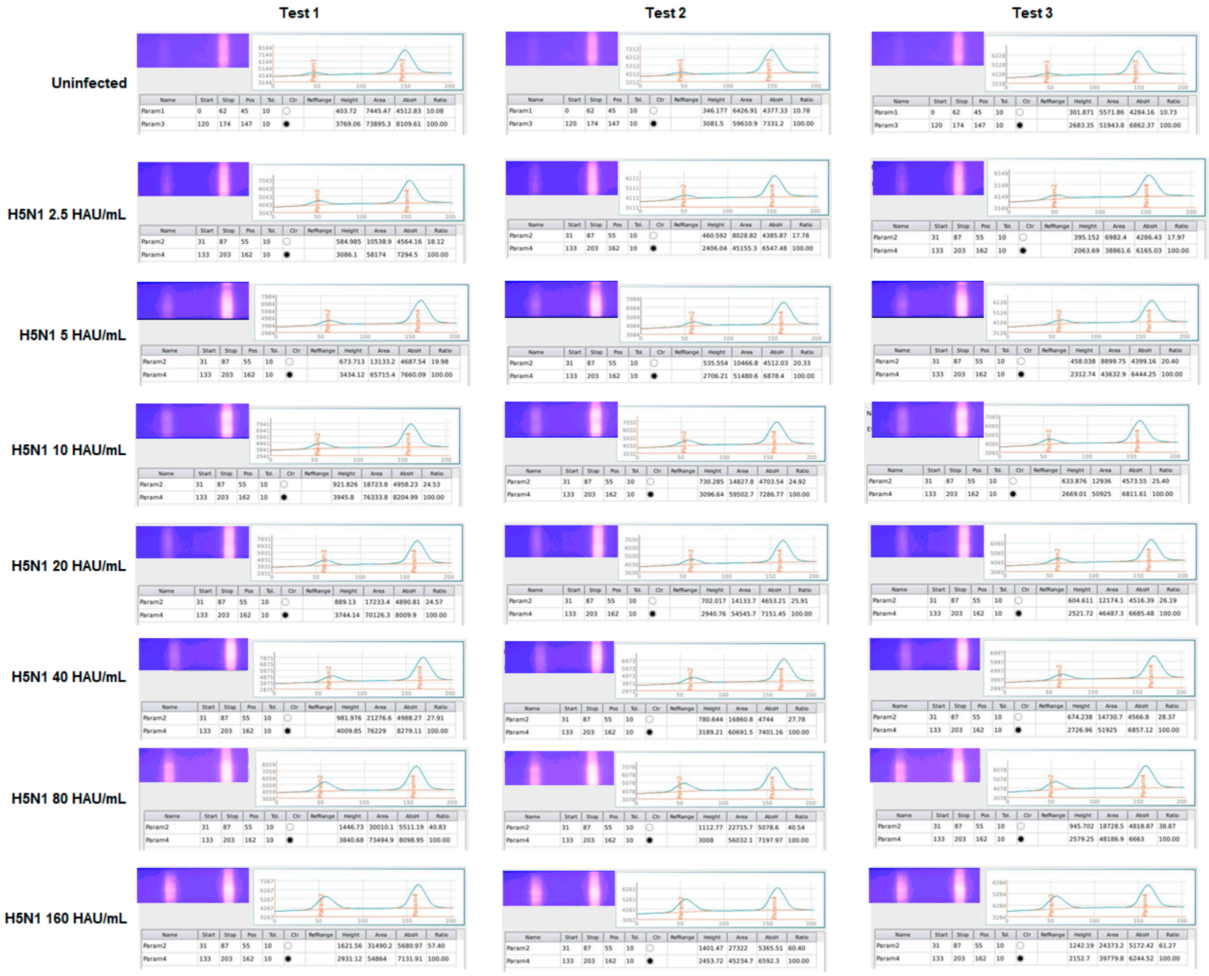
Test 1
Test 2
Test 3
Uninfected
H5N1 2.5 HAU/mL
H5N1 5 HAU/mL
H5N1 10 HAU/mL
H5N1 20 HAU/mL
H5N1 40 HAU/mL
H5N1 80 HAU/mL
H5N1 160 HAU/mL

Figure S11.

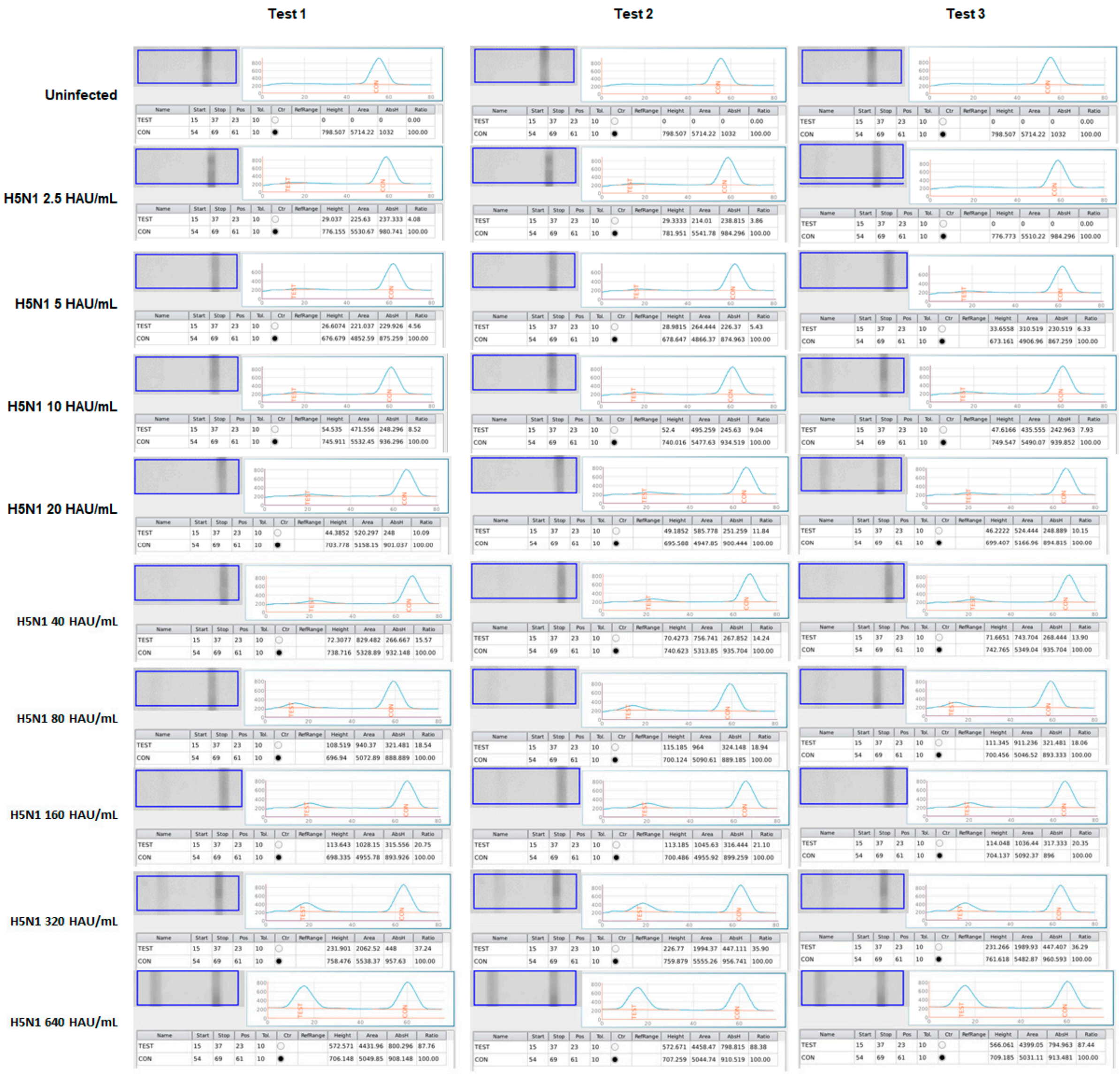

Test 1
Test 2
Test 3
Uninfected
H5N1 2.5 HAU/mL
H5N1 5 HAU/mL
H5N1 10 HAU/mL
H5N1 20 HAU/mL
H5N1 40 HAU/mL
H5N1 80 HAU/mL
H5N1 160 HAU/mL
H5N1 320 HAU/mL
H5N1 640 HAU/mL

Figure S12.

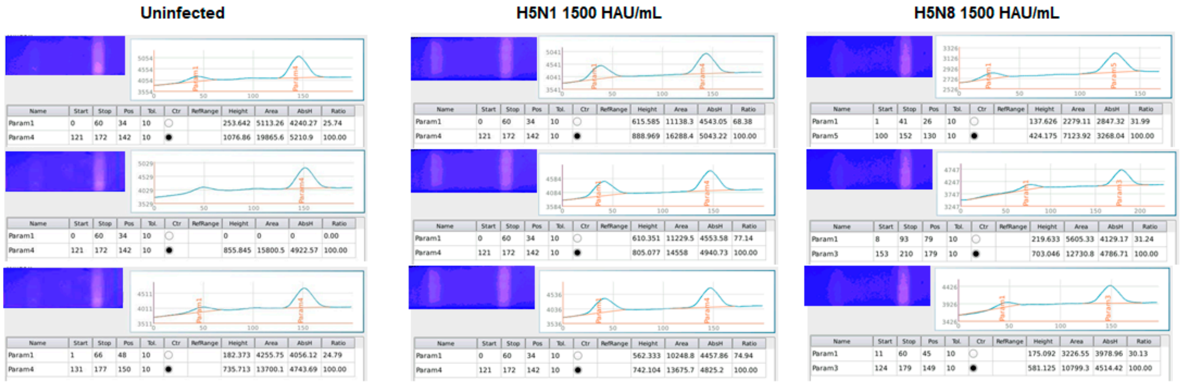

Figure S13.

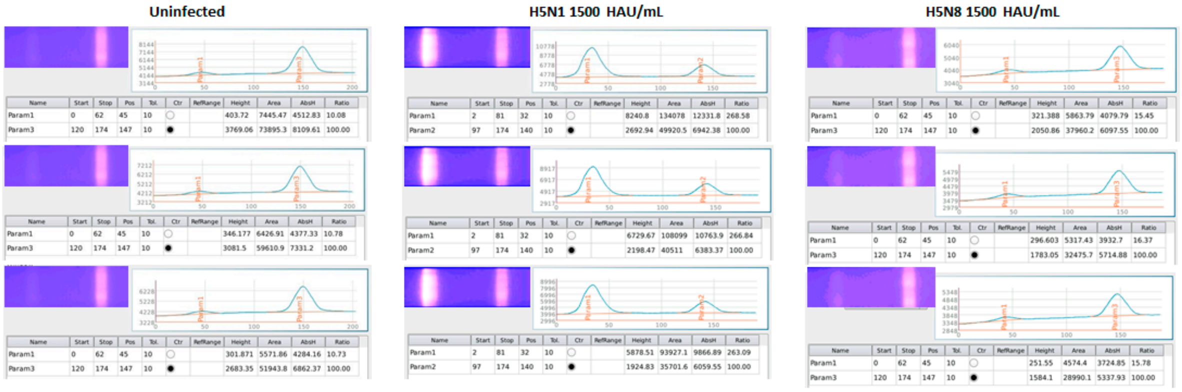

Figure S14.

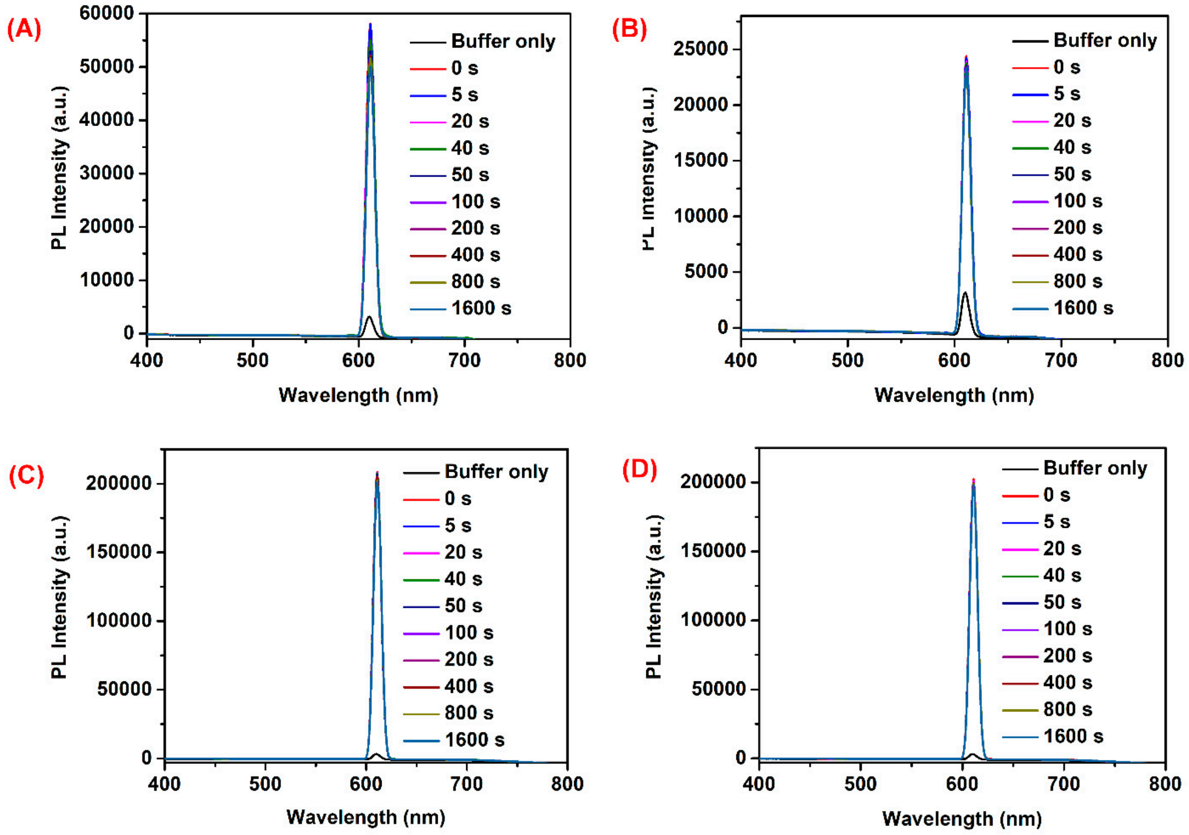

(A)
PL Intensity (a.u.)
60000
50000
40000
30000
20000
10000
0
400
500
600
700
800
Wavelength (nm)
Buffer only
0 s
5 s
20 s
40 s
50 s
100 s
200 s
400 s
800 s
1600 s
(B)
PL Intensity (a.u.)
25000
20000
15000
10000
5000
0
400
500
600
700
800
Wavelength (nm)
Buffer only
0 s
5 s
20 s
40 s
50 s
100 s
200 s
400 s
800 s
1600 s
(C)
PL Intensity (a.u.)
200000
150000
100000
50000
0
400
500
600
700
800
Wavelength (nm)
Buffer only
0 s
5 s
20 s
40 s
50 s
100 s
200 s
400 s
800 s
1600 s
(D)
PL Intensity (a.u.)
200000
150000
100000
50000
0
400
500
600
700
800
Wavelength (nm)
Buffer only
0 s
5 s
20 s
40 s
50 s
100 s
200 s
400 s
800 s
1600 s

Figure S15.

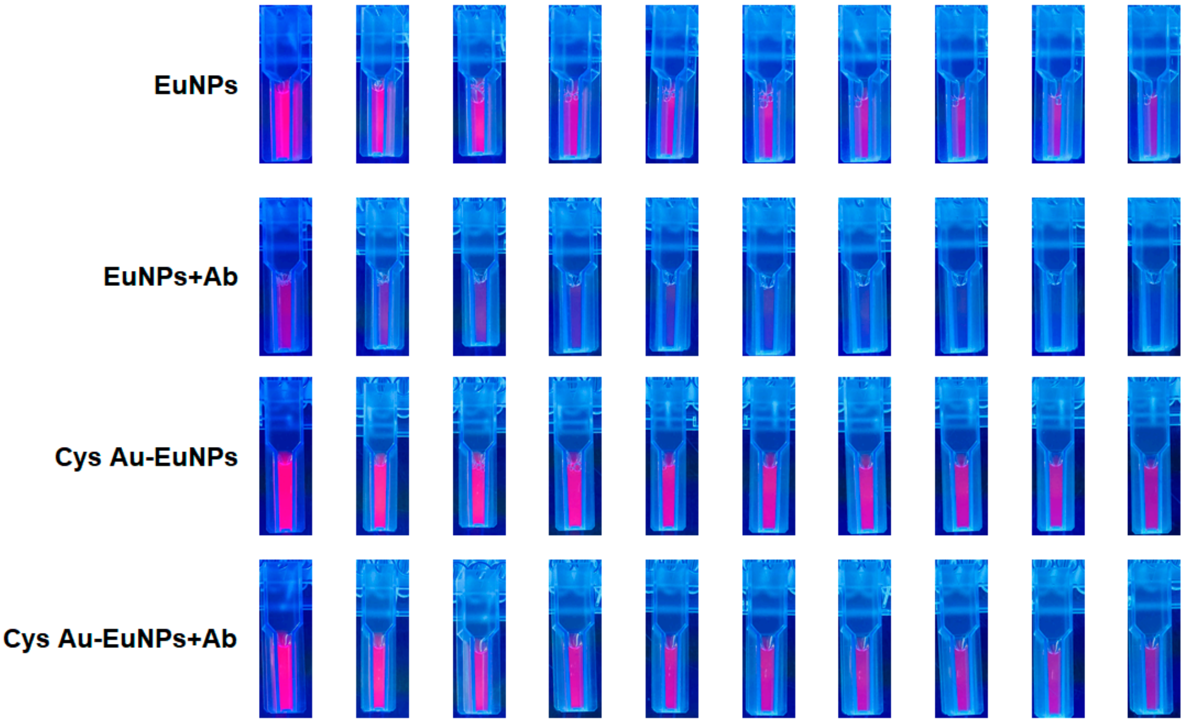

Supplement: Supplementary file 1 [file ijms-23-07957-s001.zip › ijms-1759016-supplementary.pdf]
